# Ambient fine particulate matter and ozone exposures induce inflammation in epicardial and perirenal adipose tissues in rats fed a high fructose diet

**DOI:** 10.1186/1743-8977-10-43

**Published:** 2013-08-22

**Authors:** Lixian Sun, Cuiqing Liu, Xiaohua Xu, Zhekang Ying, Andrei Maiseyeu, Aixia Wang, Katryn Allen, Ryan P Lewandowski, Lori A Bramble, Masako Morishita, James G Wagner, J Timothy Dvonch, Zhichao Sun, Xiaowei Yan, Robert D Brook, Sanjay Rajagopalan, Jack R Harkema, Qinghua Sun, Zhongjie Fan

**Affiliations:** 1 Department of Cardiology, Peking Union Medical College Hospital, Peking Union Medical College & Chinese Academy of Medical Science, Beijing, China; 2 Division of Cardiovascular Medicine, The Affiliated Hospital of Chengde Medical College, Chengde Medical College, Chengde, Hebei Province, China; 3 Department of Physiology, Hangzhou Normal University, Hangzhou, China; 4 Davis Heart and Lung Research Institute, The Ohio State University, Columbus, Ohio, USA; 5 Center for Integrative Toxicology and Department of Pathobiology and Diagnostic Investigation, Michigan State University, East Lansing, Michigan, USA; 6 School of Public Health, University of Michigan, Ann Arbor, Michigan, USA; 7 Division of Cardiovascular Medicine, University of Michigan, Ann Arbor, Michigan, USA

**Keywords:** Particulate matter, Ozone, Epicardial adipose tissue, Perirenal adipose tissue, Inflammation, Oxidative stress

## Abstract

**Background:**

Inflammation and oxidative stress play critical roles in the pathogenesis of inhaled air pollutant-mediated metabolic disease. Inflammation in the adipose tissues niches are widely believed to exert important effects on organ dysfunction. Recent data from both human and animal models suggest a role for inflammation and oxidative stress in epicardial adipose tissue (EAT) as a risk factor for the development of cardiovascular disease. We hypothesized that inhalational exposure to concentrated ambient fine particulates (CAPs) and ozone (O_3_) exaggerates inflammation and oxidative stress in EAT and perirenal adipose tissue (PAT).

**Methods:**

Eight- week-old Male Sprague–Dawley rats were fed a normal diet (ND) or high fructose diet (HFr) for 8 weeks, and then exposed to ambient AIR, CAPs at a mean of 356 μg/m^3^, O_3_ at 0.485 ppm, or CAPs (441 μg/m^3^) + O_3_ (0.497 ppm) in Dearborn, MI, 8 hours/day, 5 days/week, for 9 days over 2 weeks.

**Results:**

EAT and PAT showed whitish color in gross, and less mitochondria, higher mRNA expression of white adipose specific and lower brown adipose specific genes than in brown adipose tissues. Exposure to CAPs and O_3_ resulted in the increase of macrophage infiltration in both EAT and PAT of HFr groups. Proinflammatory genes of *Tnf-α, Mcp-1* and *leptin* were significantly upregulated while *IL-10* and *adiponectin*, known as antiinflammatory genes, were reduced after the exposures. CAPs and O_3_ exposures also induced an increase in inducible nitric oxide synthase (iNOS) protein expression, and decrease in mitochondrial area in EAT and PAT. We also found significant increases in macrophages of HFr-O_3_ rats. The synergetic interaction of HFr and dirty air exposure on the inflammation was found in most of the experiments. Surprisingly, exposure to CAPs or O_3_ induced more significant inflammation and oxidative stress than co-exposure of CAPs and O_3_ in EAT and PAT.

**Conclusion:**

EAT and PAT are both white adipose tissues. Short-term exposure to CAPs and O_3_, especially with high fructose diet, induced inflammation and oxidative stress in EAT and PAT in rats. These findings may provide a link between air-pollution exposure and accelerated susceptibility to cardiovascular disease and metabolic complications.

## Background

Adipose inflammation is a characteristic hallmark of Type II diabetes-obesity states characterized by insulin resistance. The term “metaflammation” is widely used to describe the close dependence of metabolic abnormalities to inflammation in the visceral fat tissues. The inflammation in adipose depots has been widely linked to systemic abnormalities including disturbances in glucose homeostasis, lipid abnormalities and accelerated development of cardiovascular disease. We and others have described recent findings that have linked air-pollution exposure in animal models to the development of insulin resistance and inflammation [1]. A characteristic hallmark in these studies was the development of adipose inflammation in visceral fat tissues characterized by the infiltration of innate immune cells and pro-inflammatory gene expression. Additionally, we have demonstrated a downregulation of brown adipose tissue specific genes such as uncoupling protein (*Ucp*) -1 and an upregulation of genes specific to white adipose tissue by air pollution exposure. White adipose tissue is highly adapted to store excess energy as triglycerides, while brown adipose tissue functions to dissipate chemical energy in the form of heat [2]. Recent studies have called attention to a role for epicardial adipose tissue (EAT) inflammation as an additional determinant of inflammation and susceptibility to cardiovascular disease in patients with obesity and metabolic syndrome [3]. EAT is an unusual visceral fat depot with anatomical and functional contiguity to the myocardium and coronary arteries that may serve a unique role and thus may differ from other visceral fat tissues depots [3,4]. EAT is also a source of multipotent stem or progenitor-like cell populations, which are deemed to be involved in the tissue repair and in the pathogenesis of cardiovascular disease. A growing body of evidence supports a facilitatory role for EAT inflammation in cardiovascular disease [5-8]. Clinically, the inflammation and oxidative stress of perirenal adipose tissue (PAT) may have close relation with the development of cardiovascular diseases, especially systemic hypertension. To what extent inflammation and oxidative stress in EAT and PAT is present in the context of high fructose diet and simultaneous exposure to inhaled particulates and gases has not been investigated. In this study we used a relevant rat model of high-fructose ingestion and exposed the animals to a mixture of concentrated ambient fine particulates (CAPs) and ozone (O_3_) to assess the inflammation and oxidative stress.

## Results

### Whole-body exposure data

**Figure 1:**
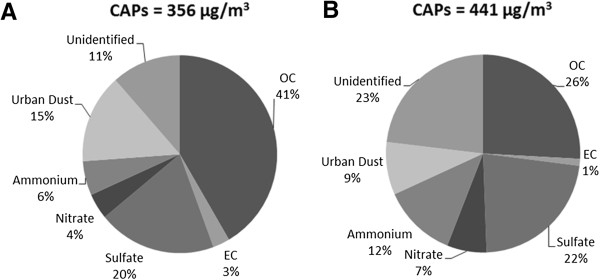
**Major compositions of CAPs during exposure periods.** Particles were collected during the exposures to CAPs alone **(A)** and CAPs + O_3_**(B)** and analyzed as described in Methods.

The average CAPs concentrations were 356 ± 261 μg/m^3^ (mean ± standard deviation; ambient =13.9 μg/m^3^) for the group exposed to CAPs alone, and 441 ± 196 μg/m^3^ (ambient = 12.5 μg/m^3^) for CAPs and O_3_ co-exposure. The average chemical composition of CAPs during the exposure periods is shown in Figure 1. As shown, the CAPs constituents were dominated by organic carbon and sulfate, which is typical for the summer months at this monitoring site and in much of the upper Midwest [9]. Average O_3_ concentrations of 0.485 ± 0.042 ppm were achieved for the group exposed to O_3_ alone, with a concentration of 0.497 ± 0.030 ppm generated during CAPs and O_3_ co-exposure.

### Body weight measurement

Table 1 illustrates the changes in body weight of the rats before and after the exposure to the CAP_S_ and O_3_ in ND and HFr groups, which had no significant differences (*p* > 0.05). In the current study, there was 10%-15% difference in body weight before and after the exposures. The “before exposure” body weight was measured in the laboratories at MSU before transporting rats to the field site seven days before exposures. Contributing factors to body weight changes include the stresses from being transported to the exposure facility and the actual exposures per se, along with no access to chow and water during the 8 h-exposures per diem. Rats were fasted an additional 3–4 hours after the last exposure during transportation to the MSU laboratories.

**Table 1:** **Body weight changes before and after exposure to CAPs/O**_**3 **_**(gram, mean ± SEM)**

**Diet &exposure**	**Baseline**	**After normal or high -fructose diet**	**After exposures**
ND-AIR	419.00 ± 11.96	570.88 ± 20.95	517.63 ± 19.53
ND-CAPs	433.63 ± 7.26	525.75 ± 16.86	503.88 ± 13.37
ND-O_3_	439.63 ± 8.05	537.00 ± 12.69	523.88 ± 13.67
ND-CAPs + O_3_	409.50 ± 12.90	546.50 ± 16.07	492.75 ± 17.45
HFr-AIR	394.86 ± 8.16	545.00 ± 14.86	507.29 ± 14.73
HFr-CAPs	436.00 ± 11.95	561.00 ± 22.02	527.25 ± 24.31
HFr-O_3_	443.25 ± 10.81	558.25 ± 14.67	531.75 ± 16.60
HFr-CAPs + O_3_	393.14 ± 9.39	531.57 ± 18.60	516.29 ± 23.03

Notes: *ND* normal diet, *AIR* ambient air, *CAPs* concentrated ambient fine particulates, *O*_*3*_ ozone, *HFr* high-fructose diet, N = 7–8.

### Characteristics of adipose tissues

#### H&E staining and TEM analysis of in situ mitochondria

**Figure 2:**
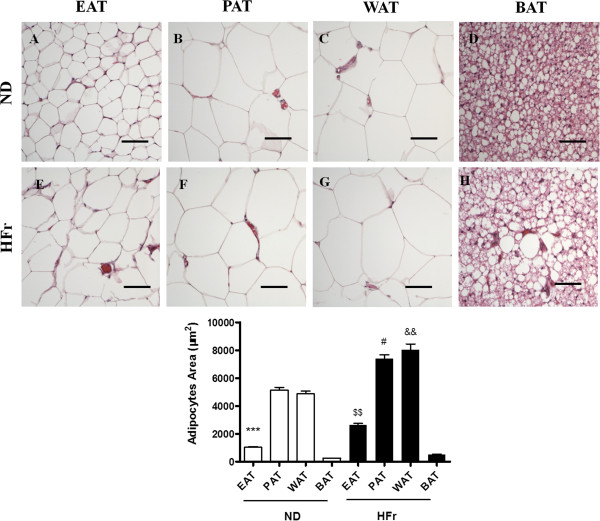
**Morphological characteristics of different adipose tissues by H&E staining.** Epicardial fat (EAT), perirenal fat (PAT), visceral fat (WAT) and interscapular fat (BAT) were from the rats fed a normal diet (ND, **A-D**) or high fructose diet (HFr, **E-H**). Adipocyte area was calculated from 100 adipocytes in each rat. Scale bars represent 100 μm. ^***^p < 0.001 *vs*. ND-PAT, ND-WAT or ND-BAT; ^$$^*p* < 0.01 *vs*. ND-EAT; ^#^*p* < 0.05 *vs*. ND-PAT; &&*p* < 0.01 *vs*. ND-WAT. N = 4.

**Figure 3:**
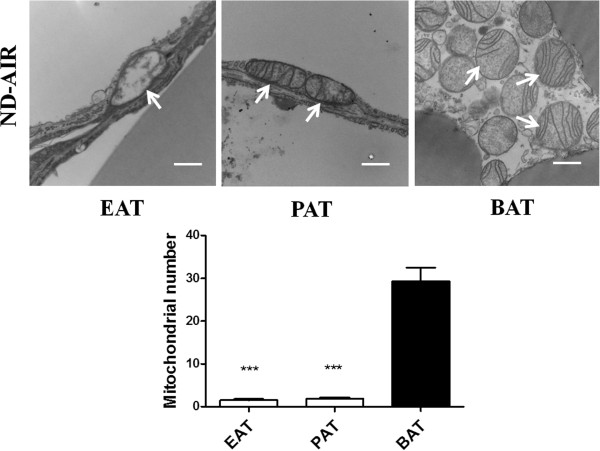
**Mitochondrial numbers in different adipose tissues.** Epicardial fat (EAT), perirenal fat (PAT) and interscapular fat (BAT) were from the rats fed a normal diet (ND) and exposed to the AIR by transmission electron microscopy (TEM). The arrows point to the mitochondria and the area of which was calculated from 6 images in each rat. Scale bars represent 500 nm. ^***^p < 0.001 *vs*. BAT. N = 4.

To determine the morphology of the adipocytes and to clarify whether the EAT and PAT demonstrates white or brown adipose characteristics, we performed H&E staining and TEM in EAT and PAT, and found that the adipocytes of EAT (Figure 2A, 2E) and PAT (Figure 2B, 2F) were similar in shape to those of visceral fats (WAT) (Figure 2C, 2G) but quite different from interscapular fat (BAT) (Figure 2D, 2H). The area of adipocytes of EAT (1,029.0 ± 43.0 μm^2^), PAT(5,133.0 ± 208.3 μm^2^) and WAT (4,870.0 ± 197.4 μm^2^) in the ND groups was much larger than that in BAT (248.1 ± 8.1 μm^2^) (*p* < 0.001). The adipocytes of EAT, PAT and WAT had single and eccentric nuclei and unilocular droplets, while BAT had multiple and central nuclei and plurilocular droplets. In addition, adipocytes in EAT were much smaller than those in PAT and WAT (*p* < 0.001). Of note, adipocytes of WAT were hypertrophic in response to HFr feeding (Figure 2G), which was also found to certain extent in EAT (Figure 2E) and PAT (Figure 2F). Figure 3 demonstrated that there were less mitochondria in EAT and PAT than in BAT (*p* < 0.001).

#### WAT and BAT specific gene expressions in adipose tissues

**Figure 4:**
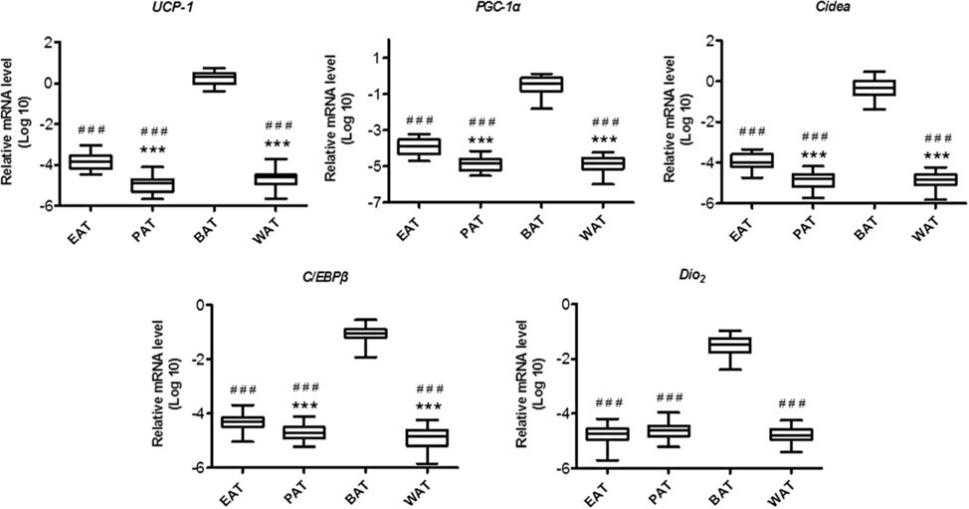
**Brown adipose-specific gene expression in various adipose tissues.** Gene expressions in epicardial fat (EAT), perirenal fat (PAT), visceral fat (WAT) and interscapular fat (BAT) were measured by real-time PCR. ^###^*p* < 0.001 *vs*. BAT, *** *p* < 0.001 *vs*. EAT. N = 7–8.

**Figure 5:**
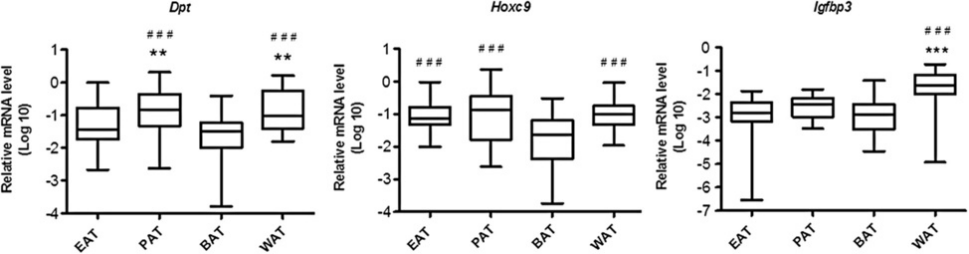
**White adipose-specific gene expression in various adipose tissues.** Gene expression in epicardial fat (EAT), perirenal fat (PAT), visceral fat (WAT) and interscapular fat (BAT) were measured by real-time PCR. **p < 0.01, *** *p* < 0.001 *vs*. EAT, ^###^*p* < 0.001 *vs*. BAT. N = 7–8.

WAT and BAT specific gene expressions in EAT and PAT were analyzed by quantitative real-time PCR. BAT specific gene expressions, such as *Ucp1, Pgc1α, Cidea, C/ebpβ* and *Dio2*, were found several hundreds of folds lower in EAT and PAT than that in BAT (*p* < 0.001, Figure 4), while the WAT specific gene expressions (*Dpt* and *Hoxc9*) in EAT and PAT were higher than that in BAT (*p* < 0.05, Figure 5). Another interesting observation in this study was that BAT specific gene expressions of *Ucp1, Pgc1,* and *Cidea* in EAT were nearly 10 folds higher than that in PAT and WAT (*p* < 0.001, Figure 4). Furthermore, the relative mRNA levels of WAT specific gene (*Dpt*) in EAT were lower than that in PAT and WAT (*p* < 0.01, Figure 5), but *Hoxc9* was much higher in EAT and PAT than that in BAT (*p* < 0.001, Figure 5). Additionally, the relative mRNA level of *Igfbp3* in WAT, also a WAT specific gene, is significantly higher than that in BAT (*p* < 0.001, Figure 5). The characteristics of different adipose tissues including EAT, PAT, WAT, and BAT are summarized in Table 2.

**Table 2:** Characteristics of adipose tissues in the rats fed normal diet

**Item**	**EAT**	**PAT**	**WAT**	**BAT**
Color	white	white	white	brown
Quantity	meager	less abundant	abundant	meager
Vascular system	+	+	+	+++
Cell morphology	large unilocular cell	large unilocular cell	large unilocular cell	small multilocular cells
nucleus	round and eccentric	round and eccentric	round and eccentric	flat and central
Mitochondria number	meager	meager	meager	abundant
Mitochondria cristae	meager	meager	meager	abundant
*Dpt*	+++	++++	++++	+
*Hoxc9*	+++	++++	++++	+
*Igfbp3*	+++	+++	+++	+
*UCP1*	++	+	+	++++
*PGC-1α*	++	+	+	++++
*Dio2*	+	+	+	+++
*C/EBPβ*	+	+	+	+++
*Cidea*	++	+	+	+++

### BAT and WAT specific gene expressions in EAT and PAT after exposure to CAP_S_ and O_3_

#### BAT specific gene expression alteration

**Figure 6:**
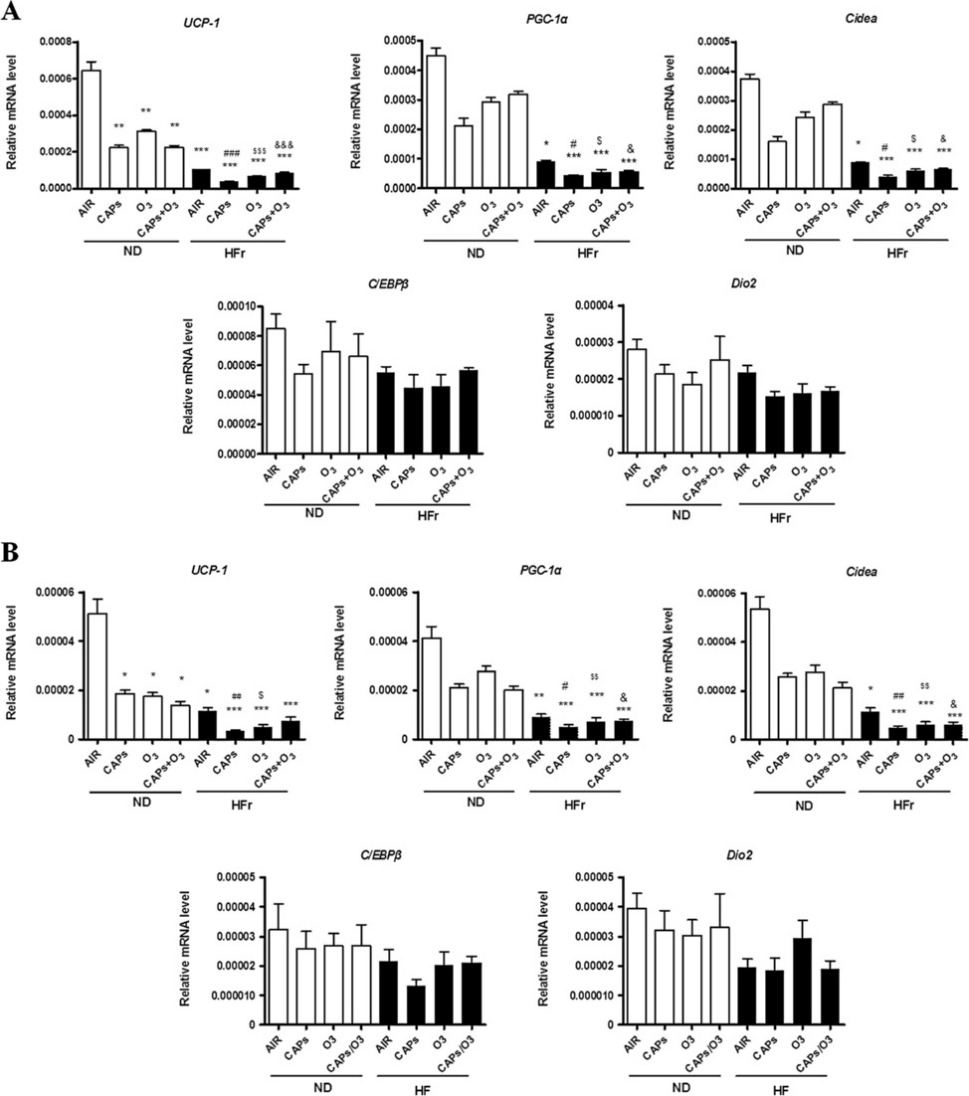
**Alteration of brown adipose specific gene expression in response to exposure to CAPs + O**_**3 **_**in EAT and PAT.** Epicardial fat (EAT, **A**) and perirenal fat (PAT, **B**) were from the rats fed a normal diet (ND) or high-fructose diet (HFr). ^*^p < 0.05, ^**^p < 0.01, ^***^p < 0.001 *vs*. ND-AIR; ^#^*p* < 0.05, ^##^*p* < 0.01, ^###^*p* < 0.001 *vs*. ND-CAPs; ^$^*p* < 0.05, ^$$^*p* < 0.01,^$$$^*p* < 0.001 *vs*. ND-O_3_; &*p* < 0.05,&&&*p* < 0.0001 *vs*. ND-CAPs + O_3_. N = 7–8.

To test if exposure to CAPs and O_3_ led to the gene expression alteration in EAT and PAT, we performed quantitative real-time PCR analysis. We found that HFr diet and dirty air exposure had synthetic interaction on the gene changes of *Ucp1,Pgc1α* and *Cidea* in EAT and PAT (Figure 6). Figure 6 illustrates that BAT-specific gene levels of *Ucp1* was significantly down-regulated in ND groups in EAT and PAT (*p* < 0.05), while *Pgc1α* and *Cidea* were not significantly down-regulated in ND groups. However, the genes expression of *Ucp1,Pgc1α* and *Cidea* were all markedly decreased, both in EAT and PAT, in HFr groups after the exposure of CAPs and O_3_ (*p* < 0.001, Figure 6A, 6B). Specifically, HFr-CAPs group showed the lowest BAT-specific gene expressions compared with the other 7 groups (Figure 6A, 6B). As shown in Figure 6, the BAT-specific gene expression alterations had similar trend in PAT as in EAT, except that *Ucp1,Pgc-1α* and *Cidea* expressions were about 10 folds lower in PAT than that in EAT. As to *C/Ebpβ*and *Dio2 genes* expression, there were no significant difference in EAT and PAT in both ND and HFr groups after the exposures (*p* > 0.05, Figure 6).

#### WAT specific gene expression alteration

**Figure 7:**
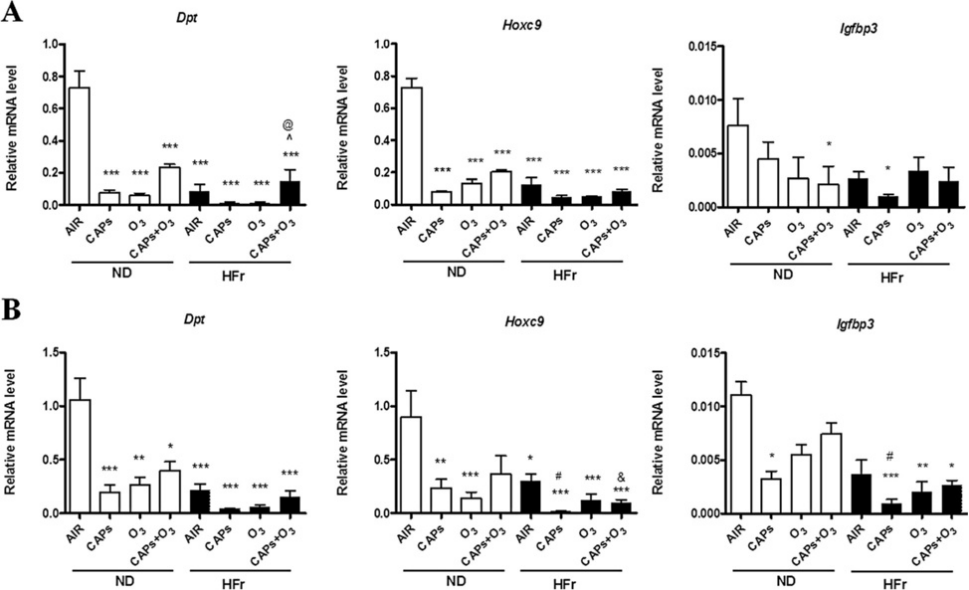
**Alteration of white adipose specific gene expression in response to exposure to CAPs + O**_**3 **_**in EAT and PAT.** Epicardial fat (EAT, **A**) and perirenal fat (PAT, **B**) were from the rats fed a normal diet (ND) or high-fructose diet (HFr). ^*^p < 0.05, ^**^p < 0.01, ^***^p < 0.001 *vs*. ND-AIR; ^#^*p* < 0.05 *vs*. ND-CAPs; &*p* < 0.05 *vs*. ND-CAPs + O_3_; ^@^*p* < 0.05 *vs*. HFr-CAPs,^ *p* < 0.05 *vs*. HFr -O_3_.N = 7–8.

It was shown that HFr diet and dirty air exposure had synthetic interaction on the gene changes of *Dpt* and *Hoxc9* in EAT and PAT (Figure 7). As shown in Figure 7, *Dpt* and *Hoxc9* expressions were significantly decreased after exposure to CAPs and/or O_3_ in EAT (Figure 7A) and PAT (Figure 7B) in the rats, especially in HFr groups (*p* <0.01). As to *Igfbp3* expression, there was less significant downregulation in EAT and PAT in HFr-CAPs groups (*p* < 0.05, Figure 7). In addition, *Dpt* gene level did not show significant decrease in the co-exposure of CAPs and O_3_ vs. CAPs or O_3_ in HFr groups in EAT (*p* < 0.05, Figure 7A).

### Systemic inflammation and oxidative stress in EAT and PAT

#### Adiponectin levels

We performed the ELISA to measure adiponectin levels in the supernatants of EAT and PAT. As shown in Table 3, there was no significant difference of the adiponectin levels among the eight groups in either EAT or PAT after different exposures.

**Table 3:** Adiponectin concentrations in adipose tissues after the exposures

**Adipose tissue**	**Diet &exposure**	**Value, ng/mL**		
EAT	ND-AIR	55.54	±	3.57
	ND-CAPs	58.38	±	0.36
	ND-O_3_	56.00	±	0.85
	ND-CAPs + O_3_	67.32	±	0.75
	HFr-AIR	56.40	±	1.66
	HFr-CAPs	62.10	±	0.93
	HFr-O_3_	59.62	±	0.87
	HFr-CAPs + O_3_	55.81	±	1.69
PAT	ND-AIR	43.64	±	1.31
	ND-CAPs	44.66	±	0.96
	ND-O_3_	44.70	±	1.02
	ND-CAPs + O_3_	45.69	±	0.70
	HFr-AIR	45.81	±	1.22
	HFr-CAPs	45.50	±	0.45
	HFr-O_3_	40.66	±	3.05
	HFr-CAPs + O_3_	51.24	±	1.06

Notes: *ND* normal diet, *AIR* ambient air, *CAPs* concentrated ambient fine particulates, *O*_*3*_ ozone, *HFr* high-fructose diet, *EAT* epicardial adipose tissue, *PAT* perirenal adipose tissue, N = 7–8.

#### Macrophage infiltration

**Figure 8:**
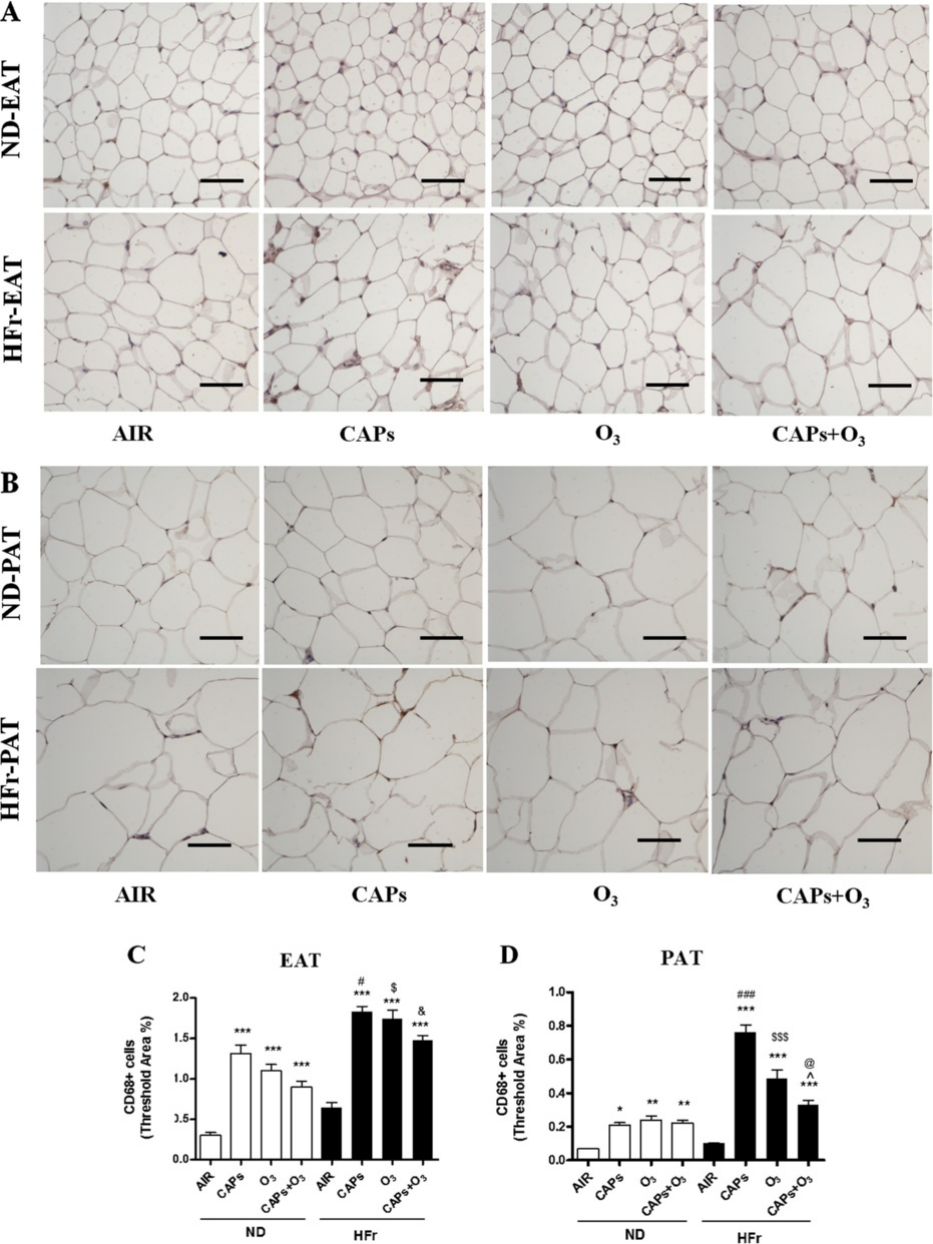
**Macrophage infiltration in response to CAPs and O**_**3 **_**exposures in EAT and PAT. A** and **B**, Representative images of immunohistochemical staining for CD68 in EAT **(A)** and PAT **(B)**. C and D, Threshold area analyses of macrophages in EAT **(C)** and PAT **(D)**. ^*^p < 0.05,^**^p < 0.01, ^***^p < 0.001 *vs*. ND-AIR; ^#^*p* < 0.05, ^###^*p* < 0.001 *vs*. ND-CAPs; ^$^*p* < 0.05, ^$$$^*p* < 0.001 *vs*. ND-O_3_;&*p* < 0.05 *vs*. ND-CAPs + O_3_; ^@^*p* < 0.05 *vs*. HFr-CAPs, ^*p* < 0.05 *vs*. HFr -O_3_. Scale bars represent 100 μm. N = 7–8.

Figure 8 illustrated representative images of CD68 (a marker of tissue macrophages in rats) staining in the adipose tissues, which showed significant increase of adipose tissue macrophages in EAT (Figure 8A, 8C) and PAT (Figure 8B, 8D) both in ND and HFr groups compared to the ND-AIR control after the exposure of dirty air (*p* < 0.001). And the more significant macrophage infiltration was found in the HFr groups than in corresponding ND groups (Figure 8, *p* < 0.05). There was no additive increase of the CD68 staining in the group of co-exposure compared with that in the groups of single exposure to CAPs or O_3_ in EAT and PAT (Figure 8).

#### Adipokine gene expression alteration

**Figure 9:**
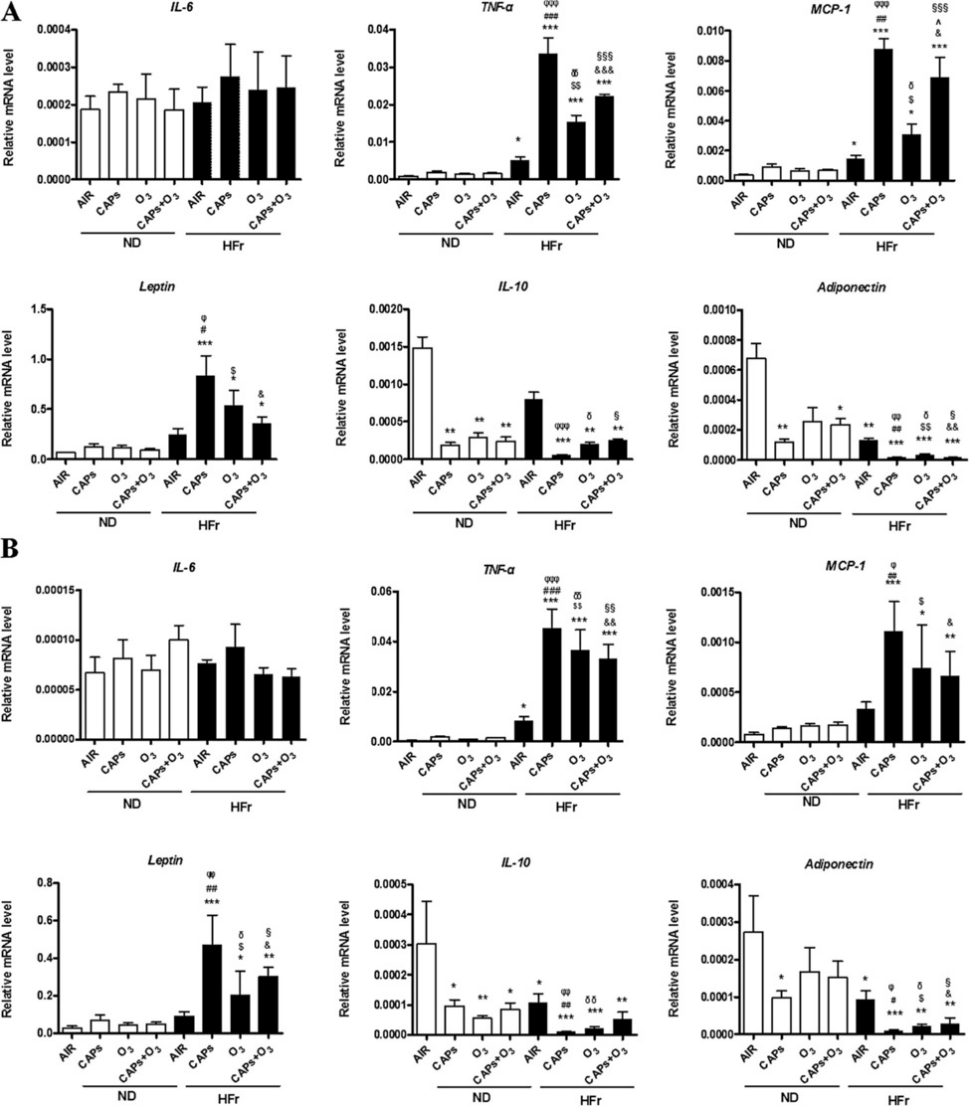
**Alteration of the adipocytokine gene expression in EAT and PAT in response to CAPs and O**_**3 **_**exposures.** EAT **(A)** and PAT **(B)** were from the rats fed a normal diet (ND) or high-fructose diet (HFr). ^*^p < 0.05, ^**^p < 0.01,^***^p < 0.001*vs*. ND-AIR; ^#^*p* < 0.05,^##^*p* < 0.01,^###^*p* < 0.001 *vs*. ND-CAPs; ^$^*p* < 0.05, ^$$^*p* < 0.01 *vs*. ND-O_3_; &*p* < 0.05, &&*p* < 0.01, &&&*p* < 0.001 *vs*. ND-CAPs + O_3_; ^*p* < 0.05 *vs*. HFr -O_3_; ^φ^*p* < 0.05,^φφ^*p* < 0.001,^φφφ^*p* < 0.001 *vs*. HFr-AIR; ^δ^*p* < 0.05, ^δδ^*p* < 0.01, ^δδδ^*p* < 0.001 *vs*. HFr- AIR; ^§^*p* < 0.05,^§§^*p* < 0.01, ^§§§^*p* < 0.001 *vs*. HFr-AIR. N = 7–8.

To explore the inflammatory gene changes in response to the exposures, we examined the mRNA by quantitative real-time PCR. As shown in Figure 9, *Tnf-α, Mcp1* and *leptin* expressions were significantly up-regulated in response to the both co-exposure and single-exposure to CAPs and O_3_ in EAT (Figure 9A) and PAT (Figure 9B) in the HFr groups (*p* < 0.05), although no significant alteration was observed in the ND groups (*p* > 0.05, Figure 9A, 9B) and no elevation in *IL-6* gene expression was found in response to the exposures. Interestingly, the HFr-CAPs group demonstrated the highest *Tnf-α, Mcp1* and *leptin* mRNA levels among all the groups (Figure 9A, 9B). Additionally, the mRNA levels of *IL-10* and *adiponectin* in EAT (Figure 9A) and PAT (Figure 9B) were down-regulated in response to the CAPs and /or O_3_ exposures, especially in the HFr group, compared to the corresponding ND-groups (Figure 9, *p* < 0.05). No significant increase of the inflammation response was found in the co-exposure of CAPs and O_3_ vs. single exposure to CAPs or O_3_ in EAT and PAT (Figure 9).

#### iNOS expression

**Figure 10:**
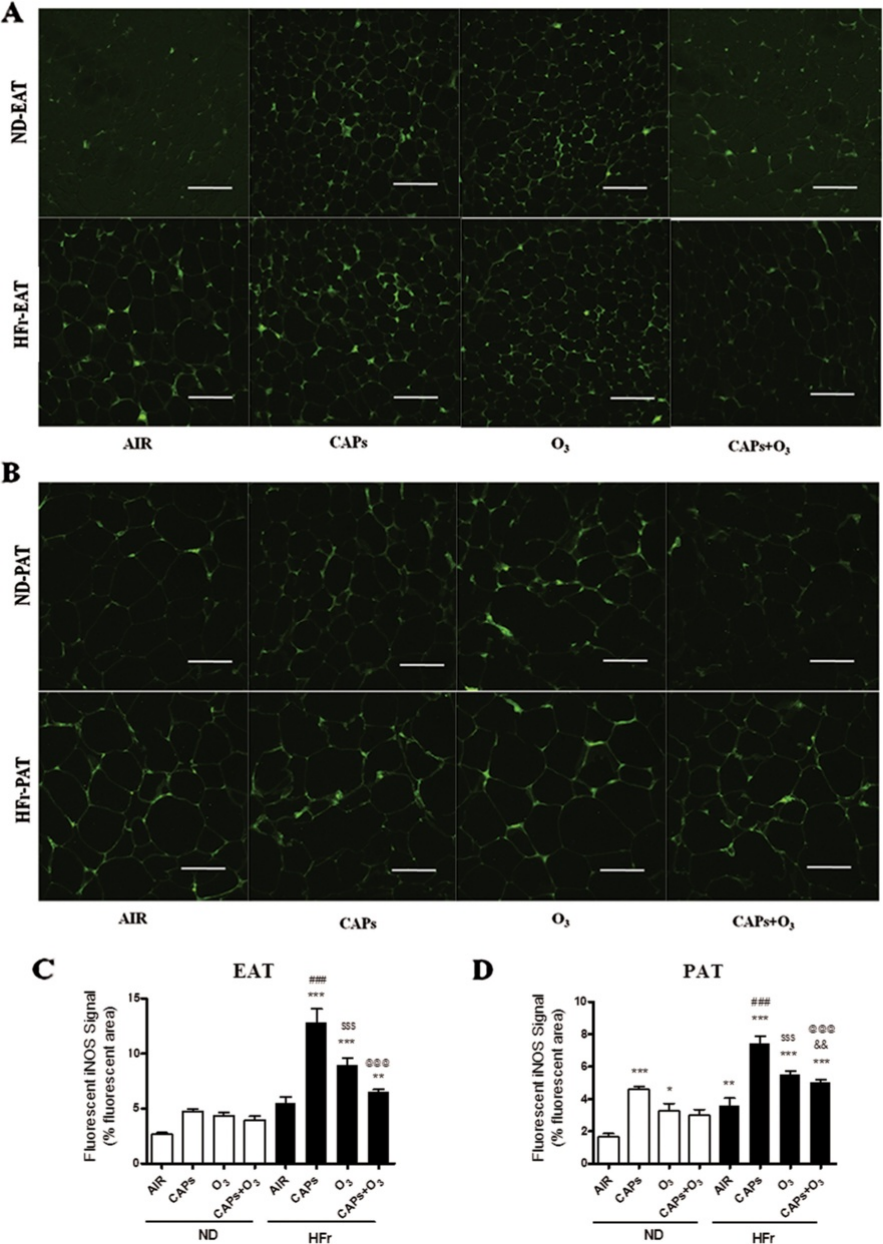
**Alteration of iNOS immunofluorescent signals in EAT and PAT in response to CAPs and O**_**3 **_**exposure. A** and **B**, Representative iNOS immunofluorescent staining photomicrographs of EAT **(A)** and PAT **(B)**. **C** and **D**, iNOS staining signals were quantified by the percentage of green immunofluorescence areas in 5 random fields per section in EAT **(C)** and PAT **(D)**.^*^p < 0.05, ^**^p < 0.01, ^***^p < 0.001 *vs*. ND-AIR; ^###^*p* < 0.001 *vs*. ND-CAPs; ^$$$^*p* < 0.001 *vs*. ND-O_3_; &&*p* < 0.01 *vs*. ND-CAPs + O_3_; ^@@@^*p* < 0.001 *vs*. HFr-CAPs. N = 4.

**Figure 11:**
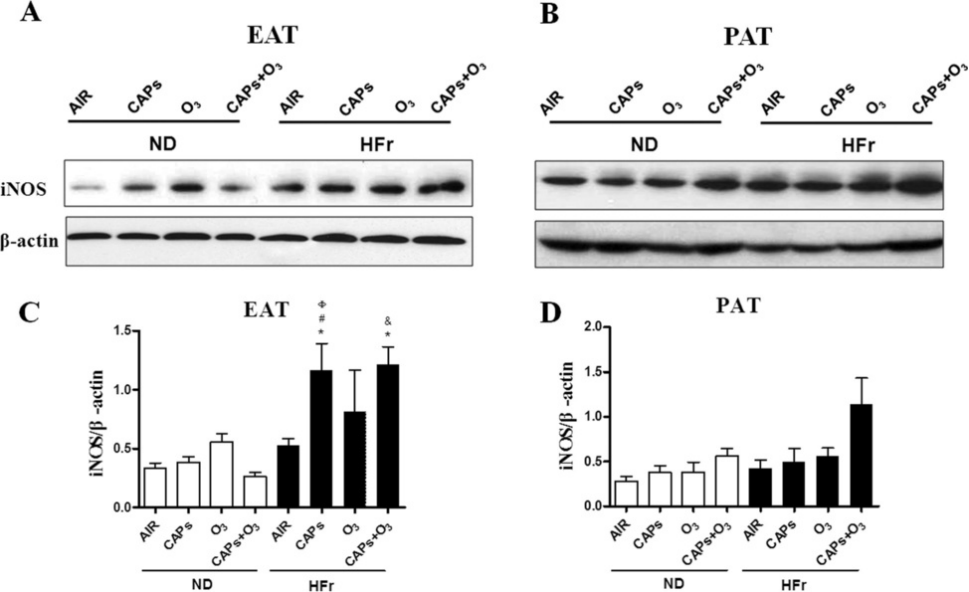
**Effect of exposure to CAPs and O**_**3 **_**on iNOS protein expression in EAT and PAT. A** and **B**, Representative bands of iNOS protein expression in EAT **(A)** and PAT **(B)**. **C** and **D**, Quantitative analyses of iNOS protein. ^*^p < 0.05 *vs*. ND-AIR, ^#^*p* < 0.05 *vs*. ND-CAPs; &*p* < 0.05 *vs*. ND-CAPs + O_3_; ^φ^*p* < 0.05 *vs*. HFr-AIR. N = 7–8.

Although nitric oxide (NO) plays an important role in various physiological processes, high concentration of NO may be cytotoxic, which can inhibit coronary artery function [10]. To evaluate whether iNOS contributed to the inflammation and oxidative stress in EAT and PAT, iNOS expression was determined by immunofluorescence. As shown in Figure 10, CAPs and/or O_3_ exposure resulted in increased iNOS immunofluorescence signal in EAT and PAT in HFr-CAPs, HFr-O_3_ and HFr-CAPs + O_3_ compared to the ND-AIR groups (*p* < 0.05). Although the iNOS immunofluorescence revealed a two folds elevation in the HFr-AIR group over the ND-AIR group, it was not statistically significant (*p* > 0.05, Figure 10C). Additionally, the strongest immunofluorescence signals were found in the HFr-CAPs groups rather than the co-exposure ones although without significant differences (*p* > 0.05, Figure 10). And significant increase of iNOS signals was observed in the HFr groups compared with the corresponding ND groups (Figure 10, *p* < 0.05). As shown in Figure 11, protein levels of iNOS were significantly elevated in the groups of HFr-CAPs and HFr-CAPs + O_3_ compared to the ND-AIR groups in EAT as measured by Western blotting. In the ND-groups in EAT and all the eight groups in PAT, however, no significant increases were found on the levels of iNOS after the exposures to CAPs and/or O_3_ (*p* > 0.05, Figure 11A-11D). Surprisingly, the highest protein levels were found in the co-exposure of CAPs and O_3_ vs. CAPs or O_3_ in EAT and PAT despite no significance difference (*p* > 0.05, Figure 11).

#### TEM in situ mitochondria alteration

**Figure 12:**
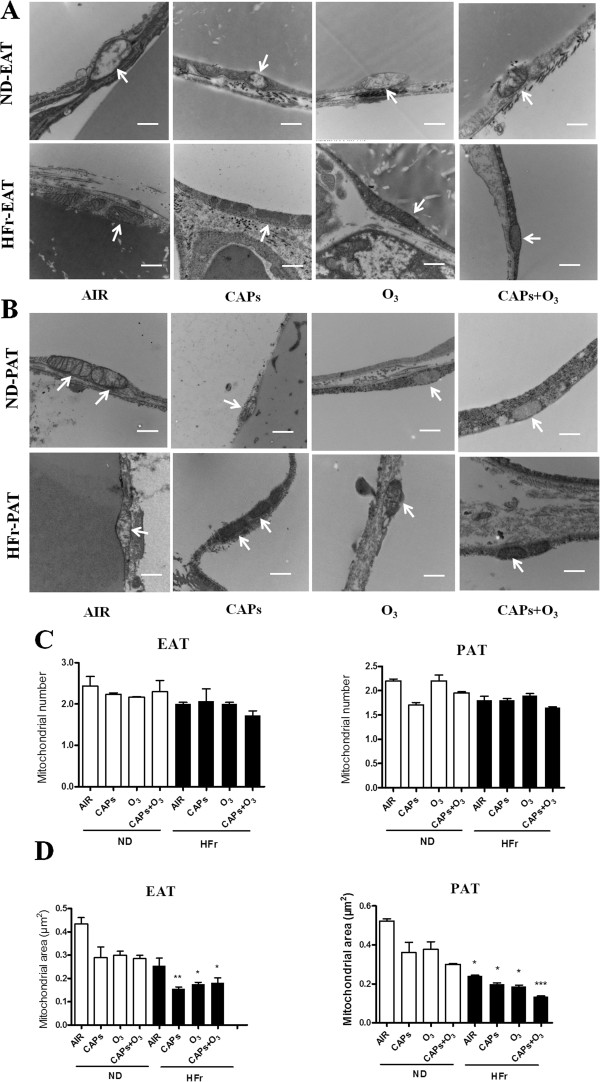
**Mitochondrial alteration in EAT and PAT by TEM in response to CAPs and O**_**3 **_**exposure. A** and **B**, Representative TEM photomicrographs of EAT **(A)** and PAT **(B)**. **C** and **D**, The analyses of mitochondrial number and area in EAT**(C)** and PAT**(D)**. Scale bars represent 500 nm. Arrows point to mitochondria.^*^p < 0.05, ^**^p < 0.01, ^***^p < 0.001 *vs*. ND-AIR. N = 4.

To explore if exposure to CAPs and O_3_ could induces mitochondria alteration in EAT and PAT, mitochondrial numbers and area were examined by TEM. Figure 12 showed representative TEM images of mitochondria in EAT (Figure 12A) and PAT (Figure 12B). Interestingly, the mitochondrial number in EAT and PAT had no significant change after the exposures (*p* > 0.05, Figure 12C). However, mitochondrial area was significantly decreased in the groups of HFr-CAPs, HFr-O_3_ or HFr-CAPs + O_3_ compared to the ND-AIR group in both EAT and PAT (*p* < 0.05, Figure 12D), although the decrease was not significant in the ND fed groups (*p* > 0.05, Figure 12D).

#### Bronchoalveolar lavage cellularity

**Figure 13:**
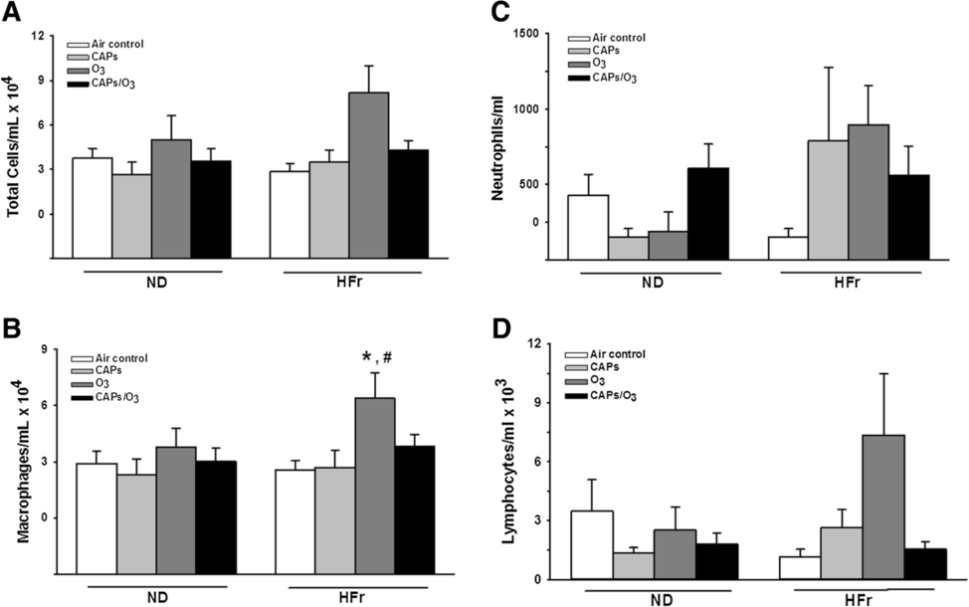
**Bronchoalveolar lavage cellularity.** Bronchoalveolar fluid contents of total cells **(A)**, macrophages **(B)**, neutrophils **(C)**, and lymphocytes **(D)** were determined in cytospin samples from rats exposed to O_3_, CAPs or CAPs + O_3_ as described in methods. * indicates significant difference from similarly expose ND rats; ^#^indicates significant difference from AIR exposed HFr rats. N = 4.

Exposure to O_3_ elicited a significant increase in macrophages in bronchoalveolar fluid in HFr-O_3_ rats (Figure 13B). In comparison, changes in concentrations of total cells, neutrophils or lymphocytes were not statistically significant (Figure 13A, 13C and 13D).

## Discussion

In this study, we investigated the morphological characteristics of various adipose tissues, which include EAT, PAT, WAT and BAT, and assessed the effects of inhalational exposures to CAPs and O_3_ on WAT and BAT specific gene expressions, as well as alterations in inflammatory gene expression in EAT and PAT in response to high-fructose feeding. To the best of our knowledge, this was the first study to evaluate the WAT and BAT specific gene alteration, especially systemic inflammatory and oxidative stress response to CAPs and O_3_ exposures in EAT and PAT in rats. Our current study showed that HFr feeding led to adipocytes hypertrophy in EAT, PAT, and WAT. EAT volume has been shown to strongly and independently reflect the fat volume of perirenal and omental visceral fat tissues lipid depots [11,12], which seems to be important in systemic inflammation [13], insulin resistance and metabolic syndrome [14]. As to the typical WAT and BAT, we have previously demonstrated the oxidative stress and changes of mitochondria and genes expression in response to the exposure of ambient fine particulates [1]. Therefore, we mainly focused on the inflammation and oxidative stress especially in EAT and PAT in the present study. We demonstrated that EAT and PAT were broadly resembling WAT based on morphology, and overall mitochondrial numbers and gene expression. Notwithstanding, EAT differed in many respects from typical WAT. Both EAT and PAT had less mitochondria than typical BAT and higher expression of WAT specific genes (*Dpt* and *Hoxc9)* while lower BAT specific gene profiles (*Ucp-1, Pgc-1α* and *Cidea),* which were approximately 1,000 folds lower than in BAT [15,16]. Interestingly, EAT was quite different from other WAT depots, with much smaller adipocytes [17] and higher mRNA levels of *Ucp-1, Pgc-1α* and *Cidea* expressions than PAT and WAT. These findings may suggest an unique role of EAT. EAT has been suggested to play a role in a variety of processes relating to preservation of myocardial form and function. For instance, it may play an athermogenic function and protect against significant excursion in temperature and protect against arrhythmias [3,18]. EAT is now recognized as a rich source of free fatty acids, a key of energy for the heart, and has been suggested to secrete a number of bioactive molecules [19,20]. Additionally, EAT also has storage function. Higher levels of both lipolysis and lipogenesis than other adipose depots confer “dual capability” of accumulating lipids for storage and also releasing them rapidly in response to demand. The latter function may explain high expressions of *Ucp-1, Pgc-1α* and *Cidea* in EAT [21].

We also found that short-term inhalational exposure to CAPs and O_3_ significantly downregulated WAT- specific genes (*Hoxc9* and *Dpt) and* BAT-specific genes (*Ucp-1, Pgc-1α* and *Cidea)* in EAT and PAT. *Hoxc9* belongs to the homeobox family of genes, and it is recognized as WAT specific marker in primary adipocyte cultures [22]. Overall, an important observation was that co-exposure to CAPs and O_3_ did not appear to potentiate co-effects with most of these genes, while HFr plus CAPs exposure was often the strongest factors pertaining to inflammation in adipose tissues. In some instances, co-exposure to CAPs and O_3_ led to less pronounced (albeit some are non-significant) inflammation in EAT and PAT than the single component exposure to CAPs or O_3_. *Dpt* is regarded as a marker for white adipogenesis and as a reference gene for the “whitening” phenomenon [1,23]. *Ucp-1* uncouples substrate oxidation and electron transport through respiratory chains from adenosine triphosphate production that results in dissipation of energy as heat and thereby playing a pivotal role in thermogenesis and protecting against reactive oxygen species (ROS) [24]. This is caused by an increased proton leakage over the inner mitochondrial membrane which dissipates the proton motive force as heat instead of adenosine triphosphate synthesis [25]. *Pgc-1α,* also a marker for BAT, induces mitochondrial biogenesis and thermogenesis [26]. The cell death-inducing DNA fragmentation factor-α-like effector (*Cidea*) family plays important roles in lipid droplet formation, and is critically involved inlipogenesisand lipolytic metabolism [27]. The downregulation of *Ucp-1,Pgc-1α* and *Cidea* in response to CAPs and O_3_ suggests an important effect of exposure in modulating thermogenic functions and lipogenesis pathways. In this current study, we did not find significant expression alteration of *Dio2* and *C/ebpβ* (BAT specific genes) [28] and *Igfbp3* (WAT specific gene) [29] in EAT and PAT after the exposures of CAPs and O_3_. Additionally, we found there were significantly synergistic interactions of high fructose and dirty air exposure on most of the WAT and BAT specific genes regulation in the present study.

A striking finding of this study was the relatively modest effects of HFr alone on inflammatory effects in EAT and PAT. In contrast, CAPs plus the HFr diet was sufficient to potentiate inflammatory responses both in EAT and PAT. It did appear to be a significantly additive or synergistic effect of both high fructose and dirty air exposure on most inflammatory genes (albeit some are non-significant). Pro-inflammatory adipokines, such as *Mcp-1, Tnf-α,* and *Leptin,* were found to be generally upregulated with the levels being highest in response to CAPs exposure alone, while anti-inflammatory adipokines, such as *IL-10* and *Adiponectin*, were down-regulated in response to the exposures in the HFr-group rats in this study. These results suggest that CAPs and O_3_ exposures may result in a pro-inflammatory macrophages shift in EAT and PAT. Interestingly, *IL-6*, also regarded as a pro-inflammatory adipokine, was not changed in gene expression in response to the dirty air exposures in this study. This was consistent with the function of *IL-6*, which was an acute-phase responsive cytokine and might be expressed at an earlier time point [30]. Surprisingly, almost no significant increases of the inflammation and oxidative stress responses were found in the co-exposure of CAPs plus O_3_ vs. CAPs or O_3_ single exposure in EAT and PAT, except the iNOS protein level, which seemed to have a ceiling effect. Another possibility could be that the O_3_ concentrations used in this project was relatively low and it was metabolized quickly and possibly locally in the upper respiratory track that might not have substantial systemic impact [31]. The discrepancy in iNOS expression between fluorescence assay and Western blot was likely due to the different sensitivity and different binding targets of those two methods [32]. We also found significant increases only in macrophages of HFr-O_3_ rats. This response in HFr-O_3_ rats was associated with mild para-acinar accumulation of macrophages, but histological responses were otherwise unremarkable (pathology not shown). By comparison, airway neutrophils, lymphocytes and eosinophils were unaffected by exposures or diet. Therefore, airway inflammation was not associated with the adipose responses we described. The synergetic interaction of HFr and dirty air exposure on the inflammation was found in most of the experiments. The attenuation of CAP_S_ effect with O_3_ has been reported in combined exposure studies previously [33,34]. In a rat model exposed to O_3_ and diesel exhaust particles, the combined exposures of O_3_ and diesel exhaust particles had less pronounced effects, which showed elevated biomarkers of oxidative stress (hemeoxygenase-1), thrombosis (tissue factor, plasminogen activator inhibitor-1, tissue plasminogen activator, and von Willebrand factor), vasoconstriction pertaining to vascular impairments in the aorta, with the loss of phospholipid fatty acids in myocardial mitochondria, than exposure to either pollutant alone [34]. In another study with rats exposed to ambient particulate matter, O_3_, or combinations for 4 hours, both pollutants transiently increased endothelin-B receptor mRNA expression, while O_3_ decreased endothelin-A receptor mRNA levels. Pollutants and O_3_ have been hypothesized to attenuate their effects by virtue of chemical reaction or chemical modification in the ambient air prior to interacting with biological molecules inside the body [35]. However, the credible mechanisms of inhalational CAPs plus O_3_ co-exposure resulting in less conspicuous effects compared with single exposure, still are lacking but remain an area of intense research in the field of exposure toxicology.

In the current study using the Harvard-type fine particle we generated CAPs concentrations of of 300 - 400 μg/m^3^ that very high can occur near point sources of pollution (e.g. traffic), in major industrial cities in north American, or some regions in developing countries like China. Such exposures are usually episodic, unlike our current protocol of nine continuous weekdays. However in a recent CAPs study in mice where exposures were conducted longer (2 months) and to lower PM_2.5_ concentrations (98 μg/m^3^), we described similar adipose responses in mitochondria number and size, *Ucp1* expression, and BAT and WAT-specific gene expression [1], as we describe in the current study. Furthermore the total mass of exposures (c × t) between the two exposures is quite similar (26–28 mg). Thus it is possible that different pathways were initiated in each study, but they both appear to converge to induce the same adipose responses. The key finding is that exposures in the current study failed to elicit responses in healthy rats, while adipose responses only occurred in fructose-fed rats.

What are the implications of this study for myocardial and coronary disease and renal function? A limitation of this study is the lack of functional characterization in response to changes in EAT and PAT with HFr diet and exposures. Thus one can only speculate on the potential implications of this study. The possible transmural movement of macrophages from EAT into the adjacent coronary artery walls has been speculated as being involved in atherogenesis and acute coronary syndromes [36]. Indeed, substantial macrophages infiltration in coronary artery vulnerable plaques has been described in EAT that was obtained during cardiac surgery of patients with severe coronary artery stenosis [37]. The upregulation of *Mcp-1* and *Tnf-α,* which are prototypical chemokine and inflammatory triggers, extensively implicated in multiple steps of inflammation and propensity for complications in Type II DM and coronary atherosclerosis.

In summary, our study demonstrates the rapid inflammation in EAT and PAT in response to environmental exposure to inhalational toxins plus high fructose diet. Our findings may provide a link between air-pollution exposure and accelerated susceptibility to cardiovascular diseases and metabolic complications, especially for multiple synergistic risk factors.

## Conclusions

HFr feeding led to hypertrophy of adipocytes in EAT, PAT, and WAT. Short-term exposure to CAPs and O_3_, especially the single exposure to CAPs, induced inflammation and oxidative stress in EAT and PAT in rats. These findings suggest that inflammation and oxidative stress in adipose tissues may be one of the important mechanisms of air pollution exposure-induced cardiovascular diseases.

## Methods

### Animals

Eight-week-old male Sprague–Dawley rats (250–275 g) were purchased from Charles River Laboratories (Portage, MI). The rats were fed either a normal diet (ND) or high-fructose diet (HFr; 60% fructose by mass; TD.89247; Harlan Laboratories, Madison, WI). Rats fed a HFr diet for 6–8 weeks develop hypertension and insulin resistance, and therefore have been used to model metabolic syndrome in humans as detailed previously [38,39]. After 8 weeks on ND or HFr diets, rats were exposed to AIR, CAPs, O_3_, or CAPs + O_3_, for a total of 8 experimental groups: ND-AIR, ND-CAPs, ND-O_3_, ND-CAPs + O_3_, HFr-AIR, HFr-CAPs, HFr-O_3_, and HFr-CAPs + O_3_ (n = 7–8 per group). Inhalation exposures were conducted 8 h/day for 9 days over 2 weeks (Mon-Fri; Mon-Thu). All rats were sacrificed 24 h after the last exposure in a laboratory at Michigan State University (MSU) in East Lansing, MI. This investigation conformed to the Guide for the Care and Use of Laboratory Animals by the US National Institutes of Health (NIH Publication No. 85–23, revised 1996), and the study protocols were approved by the Institutional Animal Care and Use Committee of MSU, an AAALAC accredited institution.

### Exposures and tissue collection

Inhalation exposures by whole-body were conducted in AirCARE 1, a mobile air research laboratory temporarily located in the parking lot of Salinas Elementary School in Dearborn, MI during the summer of 2011. This location is a stationary air pollution monitoring site run by the Michigan Department of Environmental Quality. This site continues to have some of the highest annual airborne concentrations of PM_2.5_ in the state of Michigan, and is located within 5 km of iron/steel production facilities, a coke oven, oil refinery, sewage sludge waste incinerator, and a coal-fired power plant. Thus, the PM_2.5_ at this site is impacted by multiple industrial pollutant emission point sources [9].

CAPs were generated from ambient PM_2.5_ using a Harvard-type fine particle concentrator and whole body animal exposures were conducted in Hinners chambers. The specific details of the concentrator performance and the inhalation exposure systems within AirCARE 1 have been described previously [40,41]. O_3_ was generated using an ORECO_3_ generator (Model V1; *uv* method), and O_3_ concentration was targeted at 0.05 ppm during all exposure scenarios. Due to space constraints in the exposure chambers, exposure to CAPs + O_3_ or AIR was run at different weeks (July 25 – Aug 4) as exposure to CAPs or O_3_ alone (Aug 15–25). All of the rats were sacrificed within the 24 h after exposure (Aug 5 and Aug 26), and WAT (visceral fat- omental adipose tissue),BAT (interscapular adipose tissue), EAT and PAT were harvested and stored, respectively. In addition, the trachea was exposed and cannulated, and the heart and lung were excised *en bloc*. The bronchus to the left lung was temporarily closed with a hemostatic clamp, and 5 ml of sterile saline was instilled through the tracheal cannula and withdrawn to recover bronchoalveolar lavage fluid from the right lung lobes. A second saline lavage was performed and combined with the first.

### Characterization of CAPs

CAPs mass concentrations were determined using a microbalance (MT-5 Mettler Toledo, Columbus OH) in a temperature/humidity-controlled Class 100 clean laboratory. PM samples collected on quartz filters were analyzed for carbonaceous aerosols by a thermal-optical analyzer using the NIOSH method (Sunset Labs, Forest Grove, OR). The speciation of organic and elemental carbon (OC and EC, respectively) was accomplished through gradient heating and continuous monitoring of filter transmittance with flame ionization detection. Annual denuder/filter pack samples were analyzed for major ions such as sulfates, nitrates and ammonium by ion chromatography (Model DX-600, DIONEX, Sunnyvale, CA). Furthermore, PM samples collected on Teflon filters were wetted with ethanol and extracted in 1% nitric acid solution. Sample extracts were then analyzed for a suite of trace elements using inductively coupled plasma-mass spectrometry (ICP-MS) (ELEMENT2, Thermo Finnigan, San Jose, CA) as detailed previously [38]. To estimate the contribution of urban dust in southwest Michigan, we used the equation 1.89*Al + 1.4*Ca + 1.43*Fe + 2.14*Si, where Si is estimated by K/0.15 [42].

### Enzyme-linked immunosorbent assay (ELISA)

EAT and PAT tissues (~50 mg wet weight) were incised into small pieces and immersed into 0.6 mL cell culture media, respectively, then incubated at 37°C overnight. The supernatant of the media was collected separately and stored at −80°C for the analysis of adiponectin. The adiponectin levels were determined using an adiponectin quantification kit (ELISA Kit, AdipoGen, San Diego, CA) following the manufacturer’s instructions.

### Quantitative real-time polymerase chain reaction (PCR)

Total RNA was extracted with Trizol reagent (Invitrogen, Grand Island, NY) from EAT, PAT, WAT and BAT according to the manufacturer’s instructions. Total RNA was then converted into cDNA using a High Capacity cDNA Reverse Transcription Kit (Applied Biosystems, Foster City, CA). The quantification of gene expression was determined by real-time PCR. All reactions were performed under the same condition: 50°C for 2 min, 95°C for 10 min, 40 cycles of 95°C for 15 s, and 60°C for 1 min. The primers for rat uncoupling protein 1 (*Ucp-1*), peroxisome proliferative activated receptor, gamma, coactivator 1 alpha (*Pgc-1α*), type 2 iodothyronine deiondinase (*Dio2*), CCAAT enhancing binding protein β (*C/ebpβ*), cell death-inducing DNA fragmentation factor-like effector A (*Cidea*), dermatopontin *(Dpt),* homeobox C9 *(Hoxc9),* insulin-like growth factor binding protein 3 (*Igfbp3*), *leptin,* tumor necrosis factor-α (*Tnf-α*), monocyte chemotactic protein-1(*Mcp-1*), interleukin (*IL*)-*6*, *Adiponectin*, *IL-10*, and *β-actin* were shown in Table 4. *β-actin* was used as the house-keeping gene, and all data were presented as relative mRNA levels.

**Table 4:** Primers used for real-time PCR

**Gene**	**Forward oligonucleotides (5’ - 3’)**	**Reverse oligonucleotides (5’ - 3’)**	**Product size (bp)**
*Ucp-1*	TCAACACTGTGGAAAGGGACGACT	TCTGCCAGTATGTGGTGGTTCACA	119
*Pgc-1α*	ACGAAAGGCTCAAGAGGGACGAAT	CACGGCGCTCTTCAATTGCTTTCT	108
*Dio2*	TGAGGTTAAGAAGCACCGGAACCA	CGTTGGCATTATTGTCCATGCGGT	122
*C/ebpβ*	TGATGCAATCCGGATCAAACGTGG	TTTAAGTGATTACTCAGGGCCCGGCT	97
*Cidea*	ACCATCTGTTACAGGCTGTGGGAT	AAAGCTCTTGAAAGGCCCATCTGC	124
*Dpt*	TCCACAGTTTGAGACACATCCGGT	AGAATGCACTTTCATGCTCCACGG	126
*Hoxc9*	ACGCTGGAACTGGAGAAGGAGTTT	TTTGACCTGCCGCTCAGTGAGATT	102
*Leptin*	ATGTCACCTTGCTTTGGAAGCCAC	ATGTCCCATTGTGGGCAGTACGAT	96
*Igfbp3*	ACAAGGACCTTCCTTTGTCAGGCA	AAGTCATCCGGAGAACTAAGCGCA	113
*Tnf-α*	AGAACAGCAACTCCAGAACACCCT	TGCCAGTTCCACATCTCGGATCAT	160
*Mcp-1*	TGCTGTCTCAGCCAGATGCAGTTA	TACAGCTTCTTTGGGACACCTGCT	131
*IL-6*	GTGGAAGACAAACCATGTTGCCGT	TATTGCAGGTGAGCTGGACGTTCT	116
*Adiponectin*	ATACGATGTGCTTCCTGACTGGCT	TGTTGCCAGTTTCTGTGTGGATGC	125
*IL-10*	AGCACTGCTATGTTGCCTGCTCTT	TGACTGGGAAGTGGGTGCAGTTAT	95
*β-actin*	TGAGCGCAAGTACTCTGTGTGGAT	TAGAAGCATTTGCGGTGCACGATG	129

Notes: *C/ebpβ* CCAAT enhancing binding protein β, *Cidea* cell death-inducing DNA fragmentation factor-like effector A, *Dio2* type 2 iodothyronine deiondinase, *Dpt* dermatopontin, *Hoxc9* homeobox C9, *Igfbp3* insulin-like growth factor binding protein 3, *IL* interleukin, *Mcp-1* monocyte chemotactic protein-1, *Pgc-1α* peroxisome proliferative activated receptor gamma coactivator 1 alpha, *Tnf-α* tumor necrosis factor-α, *Ucp-1* uncoupling protein 1.

### Western blotting

EAT, PAT, WAT, and BAT were homogenized in M-PER mammalian protein extraction reagent (Thermo Fisher Scientific, Waltham, MA), incubated on ice for 30 min, followed by centrifugation at 12,000 *g* for 10 min at 4°C. The supernatants were collected and subjected to Western blotting analysis. Protein concentrations were determined by BCA assay (Bio-Rad Laboratories, Hercules, CA). Twenty micrograms of protein was separated by 6% SDS - polyacrylamide gel electrophoresis and subsequently transferred to polyvinylidene difluoride membrane. After blotting in 5% non-fat dry milk in PBS -Tween 20, the membranes were incubated with primary antibody against iNOS (Santa Cruz Biotechnology, Santa Cruz, CA) at 1:1000 dilution, followed by treatment with rabbit anti-mouse IgG-HRP antibody (Santa Cruz Biotechnology) at 1:1000 dilution. The membranes were detected with enhanced chemiluminescence, followed by exposure to X-ray. The protein bands on the films were scanned, and the bands density was quantified by densitometric analysis using NIH ImageJ software.

### Light microscopic examination

The adipose tissues of EAT, PAT, WAT and BAT were fixed in formalin, dehydrated, embedded in paraffin, and sectioned for H&E staining, which were evaluated by light microscopy. For immunohistochemical staining, the deparaffinized sections of adipose tissues (5 μm) were subjected to heat-induced antigen retrieval. The slides were dipped into 0.3% H_2_O_2_ for 10 min to quench the endogenous peroxidase, incubated in 1% BSA/PBS for 10 min, followed by overnight incubation with primary antibodies(mouse anti-rat CD68 [AbD SeroTec, Raleigh, NC]) at 4°C. Then, the slides were incubated at room temperature for 2 h with appropriate horseradish peroxidase (HRP)-conjugated rabbit anti-mouse IgG-HRP antibody (Santa Cruz Biotechnology). The staining was developed using SIGMAFAST™ 3,3’-diaminobenzidine tablet set (Sigma, St Louis, MO). The sections were counterstained with hematoxylin and examined by light microscopy. All measurements were conducted in a double-blinded manner by two independent investigators using a research microscope (Zeiss 510 META, Jena, Germany) with Metamorph V.7.1.2 software (Universal Imaging, West Chester, PA). For immunofluorescence staining, the slides were incubated at room temperature for 2 h with Alexa Fluor® 488 goat anti-rabbit IgG #4412 (Cell Signaling Technology, Danvers, MA) after incubation with primary antibody against iNOS (Santa Cruz Biotechnology). All measurements were conducted in a double-blinded manner by two independent investigators using a research fluorescence microscope (Nikon, Japan) and NIH ImageJ software.

### Transmission electron microscopy (TEM)

Fresh adipose tissues were excised into small pieces (< 1 mm^3^) and fixed with 2.5% glutaraldehyde (0.1 M phosphate buffer, pH 7.4) for 3 hours. Each specimen was post-fixed in 1% osmium tetroxide for 1 h and dehydrated through a graded series of ethanol concentrations before being embedded in Eponate 12 resin, sectioned at a thickness of 80 nm and stained by 2% aqueous uranyl acetate followed by lead citrate. The grids were then observed in a Technai G2 Spirit TEM (FEI Company, Hillsboro, OR). Quantitative analysis was carried out at a magnification of 30,000X. Ten visual fields were taken randomly by a senior electron microscopist of the Campus Microscopy and Imaging Facility (CMIF) at The Ohio State University. The average numbers and area of mitochondria from ten visual fields of EAT and PAT were analyzed via NIH ImageJ software.

### Bronchoalveolar Lavage

Total leukocytes in bronchoalveolar fluid were enumerated with a hemocytometer, and fractions of eosinophils, polymorphonuclears, macrophages, and lymphocytes were determined in a cytospin sample stained with Diff-Quick (Dade Behring, Newark, DE).

### Statistical analysis

Values were expressed as mean ± SEM unless otherwise indicated. For the monofactorial continuous variable, one-way ANOVA was performed to detect the differences between different groups. For the multiple continuous variables with Gauss distribution, two-way ANOVA was performed to detect the differences between different groups, with Bonferroni correction for multiple comparison adjustment. For continuous variables with skewed distribution, the Kruskal-Wallis test was used to detect the differences between the groups. Statistical analysis was performed using SPSS 17.0 (Chicago, IL). All tests were two-tailed, and the differences were considered statistically significant at a *p* value of < 0.05.

## Abbreviations

BAT: Interscapular fat; C/ebpβ: CCAAT enhancing binding protein β; CAPs: Concentrated ambient fine particulates; Cidea: Cell death-inducing DNA fragmentation factor-like effector A; Dio2: Type 2 iodothyronine deiondinase; Dpt: Dermatopontin; EAT: Epicardial adipose tissue; EC: Elemental carbon; HFr: High fructose diet; Hoxc9: Homeobox C9; Igfbp3: Insulin-like growth factor binding protein 3; IL-6: Interleukin-6; iNOS: Inducible nitric oxide synthase; Mcp-1: Monocyte chemotactic protein-1; ND: Normal diet; O3: Ozone; PAT: Perirenal adipose tissue; OC: Organic carbon; Pgc-1α: Peroxisome proliferative activated receptor gamma coactivator 1 alpha; Tnf-α: Tumor necrosis factor-α; Ucp-1: Uncoupling protein 1; WAT: Visceral fats.

## Competing interests

The authors declare that they have no competing interests.

## Authors’ contributions

LS, CL, XX, ZY, AM, AW, KA, RPL,LAB, MM, JGW and JTD performed the experiments and contributed to acquisition of data. ZS,LS, CL, JGW,XX, ZY, AM and AW analyzed the data and interpreted the results. KA, MM and JTD contributed to CAPs and ozone exposure of the animals. The manuscript was written by LS and revised critically by QS, JGW, SR, JRH, JTD ,RDB, ZF and XY, All authors read, corrected and approved the manuscript.
